# Generating evidence for diagnosis and therapy of RarE LIVEr disease: the RELIVE Initiative for systematic reviews and meta-analyses

**DOI:** 10.1186/s13643-022-02105-0

**Published:** 2022-11-03

**Authors:** Juri Fuchs, Anastasia Murtha-Lemekhova, Jan Pfeiffenberger, Alexander Fichtner, Patrick Günther, Niels Halama, Philipp Mayer, Daniel Hornuss, Rosa Klotz, Pascal Probst, Katrin Hoffmann, for the RELIVE Initiative

**Affiliations:** 1 Department of General, Visceral and Transplantation Surgery, University Hospital Heidelberg, Heidelberg, Germany; 2 Department of Gastroenterology and Hepatology, University Hospital Heidelberg, 69120 Heidelberg, Germany; 3 Division of Pediatric Gastroenterology, Department of Pediatrics I, University Children’s Hospital Heidelberg, Heidelberg, Germany; 4 Division of Pediatric Surgery, Department of General, Visceral and Transplantation Surgery, University Hospital Heidelberg, Heidelberg, Germany; 5 Department of Medical Oncology, National Center for Tumor Diseases (NCT), University Hospital Heidelberg, Heidelberg, Germany; 6 Division of Translational Immunotherapy, German Cancer Research Center (DKFZ), Heidelberg, Germany; 7 Department of Diagnostic and Interventional Radiology, University Hospital Heidelberg, Heidelberg, Germany; 8 Division of Infectious Diseases, Department of Medicine II, Medical Center — University of Freiburg, Freiburg im Breisgau, Germany; 9 Study Center of the German Surgical Society (SDGC), University of Heidelberg, Heidelberg, Germany

**Keywords:** Rare liver disease, Rare liver tumors, Evidence-based medicine, Systematic reviews, Meta-analysis, RELIVE

## Abstract

**Background:**

Rare liver lesions and diseases have seldomly aroused major interest of researchers. For most guidelines, presumably similar clinical conditions are pooled without detailed investigations of singularities that they present.

**Main text:**

A multidisciplinary project aiming to establish evidence-based therapies for rare liver diseases has been founded. A series of systematic reviews and meta-analyses will be the starting point for a structured development of guidelines for rare conditions of the liver affecting pediatric and adult populations. The novel approach will be focusing on case reports and small patient series with distinct rare liver diseases without pooling several presumably acceptably similar conditions. Thus, a vital resource of information will be utilized, which has been largely neglected hitherto.

**Conclusion:**

Highly specific recommendations based on highest available evidence will therefore be developed for specific conditions, advancing the individualized medicine approach for the afflicted patients.

## Background

Generating evidence for rare diseases based on clinical studies is, inevitably, difficult. As a consequence, the diagnostic and therapeutic approaches for many rare conditions rely primarily on individual experiences or guidelines developed for allegedly similar but more common diseases. Yet, the assumption of clinically relevant similarity is often unsubstantiated. This means that, frequently, current treatment recommendations for patients with rare diseases are based on the lowest level of scientific evidence [1]. The heterogeneous group of rare liver diseases and tumors is particularly affected by this shortcoming [2]. Specifically, the impact of surgical therapies and different operative techniques remains yet to be investigated. For the pediatric population, the situation is even more complex as patient numbers are lower, the reporting and publication bias of cases are high, and the implementation of clinical trials has additional ethical implications and constraints.

Reviewing the literature on various rare liver diseases, a considerable amount of case reports and small case series can be found. A few patient series from multi-institutional study registries tend to be the highest level of evidence available for most diseases, with virtually all data being retrospective. Long-term observational studies including large sample sizes are lacking, and randomized controlled trials are practically non-existent. Conducting such trials is hampered by the complexity of organizing multi-institutional and international studies, and, understandably, by the low number of eligible patients and necessity of a long study duration. So far, most studies have specifically excluded case reports and smaller series from their analyses. While there are some risks and obstacles to overcome when including case reports in reviews, they present a vital resource of information and can function as a base for generating evidence. Especially for very rare diseases, these reports are invaluable as many are only sufficiently described in case reports or series and are lacking true single entity cohort studies. Important data is lost when case reports and series are systematically ruled out. We argue that the dogma of categorically excluding smaller studies from systematic reviews and meta-analyses is unjustified. The informational benefit of these studies outweighs case report specific biases, provided certain methodological and statistical principles are applied. On the contrary, case reports often supply detailed data, and statistically robust results can be achieved, when larger number of induvial cases is accumulated and analyzed.

## Structure and aim of RELIVE

Researchers from seven medical disciplines form the RELIVE consortium, including experienced physicians, senior and junior scientists in the fields of hepatobiliary surgery, hepatology, pediatric hepatology, pediatric surgery, oncology, infectious diseases, and radiology. Thus, diseases and research questions will be viewed from different angles, providing for comprehensive and objective study results.

In light of the lack of evidence for rare liver diseases, we founded the RELIVE Initiative (RarE LIVEr Disease Initiative). The aim of RELIVE is to raise awareness of rare liver lesions and conditions and to launch a collective effort for a better understanding, diagnosis, and treatment of uncommon liver diseases. For the RELIVE Initiative, we define rare liver diseases as all conditions that affect no more than one person in 2000. The focus will be set on disease with a particular lack of evidence concerning diagnosis and treatment, with all kinds of liver conditions, be it hereditary cholestatic, metabolic hepatic diseases or benign and malign liver tumors of anatomical malformations. Researchers are motivated to write more reports and carry out studies on rare diseases despite the low number of cases. To generate higher evidence, we intend to launch a series of systematic reviews (SR) that will be predominantly based on small studies on specific lesions and afflictions of the liver in adult and pediatric populations. The applied systematic approach will be a major step towards an evidence-based treatment of rare liver tumors and diseases. Although SRs and meta-analyses of small studies bear the risk of several biases, they will nevertheless provide the highest level of evidence available. We are convinced that this will lead to a better understanding of rare liver diseases and their therapy. Based on the results of the SRs and pooled data analyses, a better comparative analysis between similar conditions or variants of tumors can be made. Furthermore, RELIVE will allow for the development of clinical recommendations and research questions for future studies. Other methods such as prospective trials and translational studies can later be initiated within the framework of the RELIVE Initiative.

To ensure the highest study quality possible, a standardized systematic approach will be applied in all RELIVE systematic reviews. All systematic reviews and meta-analyses will be reported in accordance with the latest PRISMA guidelines [3, 4] and published in peer-reviewed open access journals. As provided in the PRISMA guidelines, study protocols will be drafted for all systematic reviews and registered in the international prospective register of systematic reviews, PROSPERO [5]. All included studies will be assessed for risk of bias; case reports will be evaluated based on the tool developed by Murad et al. [6]. For cohort studies of interventions or exposures, MINORS (methodological index for non-randomized studies) will be utilized [7]. In- and exclusion criteria for the study selection will be defined for each systematic review specifically adapted to the research questions. Deviation from this approach is possible only as a result of conclusive substantiations. In order to provide clear and reliable data, analyses will focus on summary statistics and univariate analyses. In-depth multivariate regression analyses or individual patient data analyses will be applied if sufficient raw data can be extracted from publications, providing statistically robust and sound results [8]. Meta-analyses or meta-regressions will only be conducted if appropriate. This means they will be used when comparative studies or several studies with larger cohorts are available [6]. To determine the certainty of evidence, the GRADE approach will be applied [9]. In order to avoid overestimation of the statistic effects found in our analyses, the interpretation will mainly focus on setting the data basis for developing clinical guidelines and for future prospective studies, instead of giving clinical directions or presenting supposedly clear results. Inferences from these systematic reviews and meta-analyses can be used to guide decision-making in the clinical practice and will be the highest level of evidence for evaluated rare conditions.

## Conclusion

Based on the RELIVE systematic reviews, robust recommendations on diagnostic and therapeutic approaches for rare liver diseases can be developed, solidified, or altered. Moreover, specific hypotheses can be derived, which can then be tested in future trials. The long-term goal of RELIVE is to engage the scientific community in developing targeted, evidence-based diagnostic and therapeutic guidelines for rare liver diseases and tumors.

## Data Availability

Not applicable

## Abbreviations

GRADE: Grading of Recommendations Assessment, Development and Evaluation
MINORS: Methodological index for non-randomized studies
PRISMA: Preferred Reporting Items for Systematic Reviews and Meta-Analyses
RELIVE: RarE LIVEr Disease Initiative
SR: Systematic review
